# Building Bridges Instead of Putting Up Walls: Connecting the “Teams” to Improve Soccer Players’ Support

**DOI:** 10.1007/s40279-023-01887-0

**Published:** 2023-07-22

**Authors:** João Renato Silva, Martin Buchheit, Karim Hader, Hugo Sarmento, José Afonso

**Affiliations:** 1 Faculty of Sport, Center for Research, Education, Innovation, and Intervention in Sport (CIFI2D), University of Porto, Porto, Portugal; 2 HIIT Science, Revelstoke, BC Canada; 3 Laboratory of Sport, Expertise and Performance, French National Institute of Sport (INSEP), Paris, France; 4 Kitman Labs, Performance Research Intelligence Initiative, Dublin, Ireland; 5 Lille OSC, Lille, France; 6 Research Unit for Sport and Physical Activity (CIDAF), Faculty of Sport Sciences and Physical Education, University of Coimbra, Coimbra, Portugal

## Abstract

The increase in the economic value of soccer occurred in parallel with an increase in competing demands. Therefore, clubs and federations evolved to greater specialization (e.g., state-of-the-art facilities and high-profile expertise staff) to support players’ performance and health. Currently, player preparation is far from exclusively club or national team centered, and the lack of control in each player’s environment can be more prevalent than expected. For example, an elite group of professional players faces disruptions in the season club-oriented planification due to involvement in national teams. Moreover, as elite players’ financial resources grow, it is common for them to employ specialized personal staff (e.g., strength and conditioning, nutritionist, and sports psychologist) to assist in their preparation, resulting in complex three-fold relationships (i.e., club, player’s staff, national team). Although efforts have been made to improve communication with and transition from the club to the national team supervision, this new reality (club-players’ staff) may generate serious compound role-related problems and difficulties in monitoring load and training adaptation and having a unified message. Therefore, efforts must be implemented to ensure a more informed management of the players’ performance environment, where the existence and impact of these various personal staff are considered to avoid a long-term non-zero sum for all intervening parties. If left unchecked, current professional thinking may collide or overlap, potentially triggering conflict escalation and impairing athletic performance or health, especially if effective communication routes are not adequately established. Moreover, diluted personal responsibility regarding performance may ensue, resulting in decreased productivity from all involved, which may cause more harm than benefits for the player’s overall health and performance. This emerging reality calls for developing a joint working framework (i.e., between the player’s personalized support team and the clubs’ team) and better managing of a player-centered process.

## Key Points

**Table:** 

Players’ performance and health have been traditionally managed by the club’s multidisciplinary teams in consultation with the national teams’ staff when applicable, but this scenario is quickly changing.
Players may also employ specialized personal staff (e.g., strength and conditioning coaches, physios, nutritionists) to assist in their preparation. Hence the need to coordinate the efforts of the club, the national team, and the player’s staff.
If no proper communication channels are established, this may impair training monitoring and prescription and unintentionally increase the risks associated with underperforming, overreaching, and/or health-related issues in current elite sports.

## Introduction

Soccer has considerably evolved in its multifaceted forms (e.g., venue design, laws of the game, competition formats) over the last decades [1]. The exponential growth observed in the industry (e.g., fan engagement and athlete performance technology support) and the distinct hierarchy [e.g., Fédération internationale de football (FIFA), Union of European Football Associations (UEFA), National Associations] and organizational levels (international, national, and club level) was fueled by increasing match, sponsorship, and broadcasting revenues [2]. This growth was reflected in an unprecedented growth of player salaries [3–6], market values, and net transfer expenses in the last three decades [7]. For example, soccer transfer fees reached a record high of USD 7.4 billion in 2019, almost tripling the fees paid in 2012 [2]. More recently (January 2023), there was more than 14.4% of the volume of transfers, and more than 49.9% of the total value of transfer fees agreed compared with January 2022 in men’s soccer [8]. This phenomenon extended to women’s soccer, as observed in a new record high in fees and more than 30.2% in the volume of transfers between the window of January 2022 and 2023 [8]. To “fuel” this evolution, soccer government bodies introduced adjustments at different levels, such as alterations in specific game laws (e.g., Law 3—number of substitutions and Law 12—back-pass rule) [9] and competition schedules [10, 11].

**Fig. 1:**
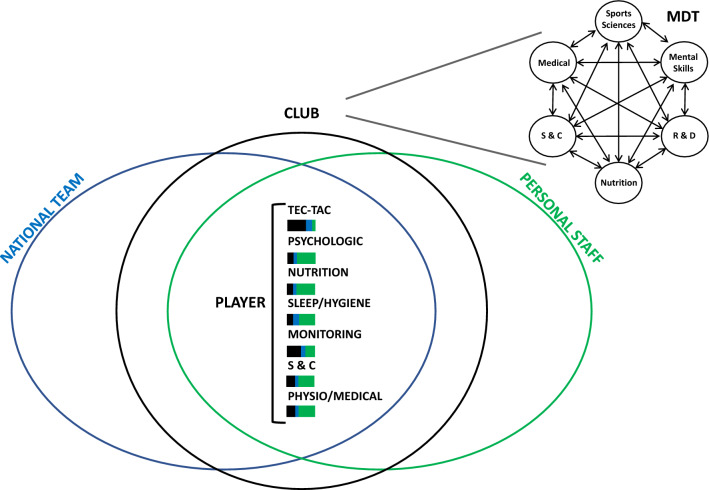
Example of the commonality of interactions between the club’s multidisciplinary team, national team, and players’ personal staff during the season (e.g., during the in-season period). An example of club multidisciplinary team is also presented (adapted from [1] to reflect communication lines and organizational challenges; (i) the role (position) of the high performance director, although not present in the figure, is critical for ensure excellent top-to-bottom management, (ii) upper right corner with double-headed arrows reflecting internal multidisciplinary team dynamics). TEC/TAC, technical-tactical; PHYSIO, physiotherapy; S and C, strength and conditioning; MDT, multidisciplinary team; R and D, research and development; colour bars represent the proportion of involvement in a specific topic. The example intends to illustrate the commonality in specific performance and health topics and the proportion of time dispended. The proportions are provided as examples and are probably player and context-specific, i.e., it may change according to the seasonal period (off-season, pre-season, and in-season normal or congested competitive period), players being selected for national team or not, and each player’s personal staff dimension, characteristics and qualities

The increase in the economic value of soccer occurred in parallel with an increase in competitive demands, such as the number of national and international competitions [10, 11] and more intense match activity profiles [12–14]. Individual players may experience around 10 consecutive weeks of a congested calendar, including domestic and international matches [10, 11]. Professional clubs are exposed to 20 + weeks of fixture congestion across a competitive season [10, 11]. Moreover, high-intensity running distance and actions increased by ~ 30% and ~ 50%, respectively, and sprint distance and the number of sprints increased by ~ 35% and ~ 85%, respectively, within specific time frames [12–14]. These changes had the burden of raising the psychological and physiological stresses placed on the athletes [15–17]. Therefore, clubs and federations evolved to greater levels of specialization to support players’ performance and health (Fig. 1). This evolution was not exclusive to the first team environment: with the extensive professionalization in clubs, the academies, and/or youth teams (e.g., the elite player performance plan by the Premier League) [18] also benefited from this more structured approach (e.g., technology support, background staff, and quality of sports facilities) [19].

However, player preparation is currently far from exclusively club centered, and the lack of coordination in each player’s environment can be more prevalent than expected. For example, an elite group of national team players may have their performance preparation and health supervision under the umbrella of two different entities throughout the season (the club’s multidisciplinary teams in articulation with the national teams’ staff). However, this scenario quickly changes (e.g., due to coaching turnover and players transfer) [20, 21]. As elite players’ financial resources grow, it is common for players to employ specialized personal staff (e.g., strength and conditioning coaches, physios, nutritionists, and sports psychologists) to assist in their preparation. This new reality results in complex two- or three-fold relationships (i.e., with club, player’s staff, and national team when applicable) [22, 23]. This results in specific areas of player support (e.g., physiological and psychological) being oriented from two (club and national team or club and player’s staff) or even three different parties (national team, club, and player’s staff, Fig. 1).

These realities challenge the organization and management of the player’s preparation, as effective performance- and health-related concerns depend strongly on the quality of the intrateam communication (i.e., within the club or the national multidisciplinary team) [24, 25] and interteam communication (club—national team—players staff). In particular, the need to articulate training and recovery processes with the player’s staff brings emerging challenges that require reflection to avoid overlapping (e.g., duplication of training practices and concurrent contents) or even contradictory interventions and prevent unnecessary interpersonal conflicts.

The above-described complexity in player preparation highlights the importance of Situational Awareness in modern soccer. According to Stanton et al. [26] Situational Awareness can be described as “a dynamic and collaborative process binding agents together on tasks” (p. 1288) and is defined as the awareness that people (e.g., players, physio, doctors, sports directors), teams, organizations, and even entire sociotechnical systems have of what is happening [27, 28]. Teams with a remarkable ability to engage with and adapt dynamically to their environment can possess a strong Situational Awareness [26]. Distributed Situational Awareness takes a systems approach to team Situational Awareness [26]. Distributed Situational Awareness has become increasingly important in fields where distributed teams and complex systems are common [27], and is an emergent property held by the overall system and is maintained and built through interactions and transactions in awareness between “agents” (e.g., tools, documents, displays) [27, 28]. Without Distributed Situational Awareness, system performance may be compromised, and systems failure may occur [28]. Further, as technology advances (e.g., artificial intelligence), the potential of Distributed Situational Awareness to improve safety, efficiency, and overall performance is likely to become even more prevalent independently of the sport [27].

This current opinion explores why and how to implement such communication and articulation to ensure win–win relationships for the player’s benefit and decrease the somewhat apparent lack of Distributed Situational Awareness among players, clubs (staff), national teams, and individual staff. With this manuscript, we hope to spark discussion and foster the development of systematic or evidence-driven approaches and frameworks to address this growing problem.

## The Within-Club Multidisciplinary Team

Initially, there was the coach and the players. The coach conducted the warm-up and the central part of the training session, analyzing the opponents and negotiating with the players. As soccer evolved, so did the challenges, and athlete-centered structures with state-of-the-art sports facilities and materials for performance optimization have been created [1]. Consequently, technical staff (assistant coach, “physical trainer,” goalkeeper coach, scouter) and medical- and health-related practitioners were included in the “family*”* (e.g., physiotherapists, sports physicians, nutritionists) [1, 21, 29]. Moreover, the contribution of sports scientists (e.g., physiologists and biomechanics) became progressively well-recognized by directors, coaches, and top professional players [1]. In the present day, a top professional team may have around 16 staff members on the pitch with the players during the warm-up [30] (and whether they have or do not have an active role is outside of the scope of the present manuscript) [31].

To increase performance and reduce injury incidence, elite clubs implement training programs, monitoring strategies, and complementary strategies to aid player recovery [16, 20, 32]. Moreover, given the specific characteristics of each player (e.g., age and profile, field position training status, injury records, lifestyle), there are significant acute and chronic inter-individual responses to training and match stimuli [33, 34]. As such, players’ training programs may differ [16, 35]. To this end, the skills of specific practitioners or specialists within the multidisciplinary team come together to overcome complex performance problems synergistically [25, 36, 37]. Consequently, the level of interaction and professional overlap between each staff member may change throughout the season according to each player’s needs (e.g., players in different stages of rehabilitation, academy players in transit for first-team training, and players returning from national teams’ commitments).

This complex dynamic can generate compound role-related problems. Current professional thinking may collide, professional overlap may exist, and ineffective communication may arise and trigger conflict escalation if some structural risk factors are not considered and effective strategies are implemented [36, 37]. Communication is critical in developing Situational Awareness in teams [38]. In this regard, information exchange is linked with high levels of Situational Awareness [39], and high levels of Situational Awareness are connected with high levels of team performance [40, 41]. On the one hand, the compatibility between agents’ Situational Awareness will determine the degree of safety and performance efficiency [28]. In contrast, the incompatibilities between agents’ Situational Awareness threaten performance, safety, and resilience [28]. For example, there is an association between the quality of the internal communication between the medical and technical staff and reduced injury burden, increased training attendance, and greater match availability [24].

Unsurprisingly, the first order of communication and organization challenge is related to the multidisciplinary team, i.e., within the club (i.e., internal communication). In fact, given the complexity of working in high-performance environments with multiple people in the same team with different philosophies and egos, we first need to control what we can control [31]. This can be achieved via well-defined communication lines, but above all, by role definition and excellent top-to-bottom management that allows collaborative work and helps build trust between staff to foster constructive criticism, ability to agree to disagree, etc. [31]. Significantly, at the moment, an effective centralization across first teams and academies can be observed in most clubs, with academy medical and performance units strategically aligned and closely integrated with their respective first team units [19].

However, other communicational and organizational challenges currently exist that make monitoring load and training adaptations considerably more demanding and dependent on the quality of the external communication process.

## Club and National Team

Although the club “owns” the player for the duration of the contract, the FIFA regulations of the status and transfer of players state: “Clubs are obliged to release their registered players to the representative teams of the country for which the player is eligible to play on the basis of his nationality if they are called up by the association concerned. Any agreement between a player and a club to the contrary is prohibited” [42]. As so, an elite group of professional players faces frequent short- (8–10 days, 4–5 times a year) and medium-term (e.g., World Cup or Confederations Cup) disruptions in the season club-oriented planification due to involvement in national teams [20, 21, 25]. Distinct training methods and monitoring systems (e.g., technologies and data collection processes) may be implemented or not even occur [20]. Every national team break disrupts the training and monitoring that the clubs implement [20].

Coaches may fear that this interruption in players’ monitoring and training will negatively impact players’ performance when returning to the club, as they may arrive over- or under-loaded (starters and non-starters, respectively) [20]. Moreover, some players may be available to compete and be included in the national team but require specific rehabilitation training protocols [20]. The national team’s commitments involve much international team travel, limiting structured training and recovery opportunities within a club-crowded competitive calendar. As such, it is paramount for clubs and national teams to communicate to anticipate problems better or conjointly solve them when they emerge properly. Otherwise, these factors may substantially increase each player’s psychological and physiological stress. Players may lose their competitive ability (e.g., players who do not compete lose the chronic load, or vice-versa, or may arrive near club competition and cannot be included in the club’s next competitive match) [43–45].

Communication is the transaction that allows awareness to be developed within a distributed team, and as a two-way exchange, it can be affected by team dynamics [46]. Since clubs and national teams may present different (sometimes antagonistic) goals at any given moment, it is fundamental to foster communication between the different multidisciplinary teams for a long-term non-zero sum for all intervenient (club-player, national team) [21]. Communication (e.g., information exchange) between these two entities is considered vital for the mitigation of injury risk and training program development [47], given the documented association between training load (internal and external) and performance [48–52] and injury in soccer [53–60]. Otherwise, reduced personal responsibility regarding performance may ensue, resulting in decreased productivity [36, 37]. This communication between the club and national team represents another communication and organization challenge that multidisciplinary teams may face given the internal and external communication requirements (intrateam and interteam communication, respectively). Experts are characterized by possessing a high level of confidence in their opinions [31, 37]. The collaborative process required can be very challenging when different ideas and experiences are present, and there is no established process to allow a softer integration [37].

Buchheit and Dupont [21] discussed possible solutions for assessing readiness to play, fitness, and monitoring training and match loads in national teams and elite clubs. McCall and colleagues [25] paved the way further when trying to establish an agreement in this context of transition from club to the national team regarding (i) what medical and physical information to collect, (ii) how to use that information, (iii) identifying challenges to the collection, and (iv) collection methods. The authors [25] found two pragmatic solutions to overcome the previous challenges: (i) access to standardized information reporting and (ii) an electronic shared database. These adoptions permit transactions (exchanges) in Situational Awareness between entities, allowing a system to develop and dynamically updated Distributed Situational Awareness [46]. Both modify their Situational Awareness as a result [46]. However, at least from our knowledge, no publications explored the pragmatic approach proposed by McCall and colleagues [25], i.e., this may be explained by the recent year of the publication. Data on players’ preparation in the clubs must be shared between the involved parties (club-players, national team). From the authors’ experience, some national teams who played in the final phase of the 2022 World Cup still completely disregarded clubs’ interests and did not share any data, while the opposite may also be valid [31]. A synthesis and adaptation of these ideas are included in the previously presented Fig. 1.

## National Teams and Players’ Staff

According to the specific competitive calendars (Europe versus South America), there are international periods (e.g., World Cup and Confederations Cup) where players may have a window of time between the terminus of the club obligations (release from the club) and the start of concentration with the national team. During this period, players may be under the umbrella of the personal staff. However, the most frequent international windows are typically 9 days, often during the club’s competitive period, wherein most scenarios have two matches [42]. Players must present themselves at the club as soon as this period ends.

Usually, national team concentrations occur away from the player’s residence (most players play in leagues other than one of their countries) and operate in a close environment (e.g., players train, eat and sleep inside the national soccer center). The time outside these training centers is just for traveling to the country where the away game will occur. During these short-term national team periods, no opportunities exist to interact physically with the players’ staff, so the exposure will be zero (as most personal staff are located where players live).

As will be discussed afterward (5.2 Why build bridges?), the degree of commonality between the club, national team, and players’ staff will depend on each of their characteristics and qualities. There may be cases where the best solution would be to centralize the information exchange in the club, i.e., when communicating with the national team, the data already includes exposure with the personal staff (e.g., training load, a program design, and rehab). Otherwise, it would be best for the player’s staff to communicate directly with the national team. We acknowledge that this process is not as simple as it may seem, especially if rules and what is valued vary between national teams/federations. In fact, some national teams/federations may only recognize player contracts with clubs and likely assume they owe no explanation to players’ staff [31]. Independently of the strategies and process adopted (e.g., the club centralizes the information versus the national team players’ staff), it should always aim to reduce the space for unclear and contradictory information (e.g., when the data from players’ staff to the national team differs from the one transmitted from the personal staff to the club), often promoting conflict and decreased performance efficiency.

While the articulation between the clubs and the national team may still be challenging, there is a potential additional issue. Indeed, players’ staff (third layer) are a relevant puzzle piece.

## Club and Players’ Staff

Players follow the same evolutionary pathway as observed at industry and institutional levels: modern players deal from an early age with professionals with backgrounds in psychology, physiology, strength and conditioning, biomechanics, and performance [1, 19]. This interaction should result in more knowledgeable players, better attuned to key performance determinants (e.g., lifestyle, recovery habits), with more significant proactive thinking and a more active role regarding relevant aspects of their career. In this regard, news in the media [61, 62] reveals players’ interests in performance analysis, training, conditioning, and health. From our experience, also frequently observed in the media [63, 64], player preparation is far from exclusively club centered, and the potential lack of supervision and/or communication in each player’s environment is of concern.

As modern elite players’ financial resources grow exponentially [4, 7], it is increasingly common for individual players to employ specialized personal staff (e.g., strength and condition, physiotherapists, nutritionists, and sports psychologists) to assist in their preparation. This reality is likely associated with the belief that having a personal staff will ensure an increase in significant performance and health, and a competitive advantage in the short- (e.g., gaining an edge over their teammates) and long-term (e.g., increasing career length).

### What Drives the Player?

Adopting a personal staff can be motivated by several factors of varying complexity. Considering player preferences, the player may wish for more personalized support [22, 23]. Athletes’ opinions and coaches’ perspectives strongly impact the effectiveness and success of players’ preparation [22, 65]. Development in the communication process between athletes, coaches, and practitioners is fundamental to improving performance-related and health-related outcomes (e.g., injury prevention, rehabilitation, nutrition, and well-being) [22, 65, 66]. Adopting a personal staff may also arise from the bond a player develops with a coach or practitioner when absent from club-organized training activities. Players usually require external services during the off-season period (e.g., to avoid detraining or performing additional rehabilitation work) [17]. During this period, the player may develop professional confidence (perhaps even some dependence) in the personal staff and may wish to extend this relationship throughout a complete season or even across significant periods of their career.

Considering the interindividual variability in response to training [67, 68] and match stimuli [33, 34], and the role of personal preferences [22, 23], having an individual staff may ensure a better-tailored program, delivering superior overall experience and results (e.g., physical, social, and psychological). For example, due to socioeconomic disparities, some clubs in leagues and countries may need more time, human resources (e.g., sports psychologist and physiologist), and facilities (e.g., technology use) to implement individualized work [69]. As a result of the previous facts, the player may seek specific support not available at the club. Even when the club has the necessary conditions, there may be a disconnect between the coach’s training philosophy and the player’s perceptions of the most beneficial practices from an individualized approach to performance and health [22]. Different beliefs across several specific themes (e.g., the proportion of general versus specific preparation, the importance of linear and multidirectional speed, the role of resistance training, and “optimal” warm-up) may result in the player needing to complement the in-club preparation with extra work.

Even when there is a perfect alignment between the player’s needs and preferences and the coaching philosophy and methods, the unstable reality of soccer may break this process apart. Dismissal of the head coach and corresponding supporting staff is a frequently occurring phenomenon in soccer [70, 71]. Some clubs may change the coaching staff more than once during the same competitive season [70, 71]. This coach turnover will likely result in constant (and arbitrary) changes in preparation models and strategies, with players having to regularly readapt, potentially impairing their ability to stabilize their performance. Interestingly, there is a link between coach turnover and increased rates of muscle strain injuries [70]. The coach carousel or turnover phenomenon may also be one of the causes that motivate players to employ specialized personal staff and so ensure a stable follow-up throughout the season(s).

Hiring personal staff may also happen because it is fashionable. As so, players may dedicate their time outside training obligations to improve, among other things, psychological aspects, specific physical characteristics (e.g., speed), or performing extra activities (e.g., massage, yoga) that they judge relevant to their health and performance, thus impacting their careers. This occurs not only during specific season moments (e.g., off-season), but may occur more or less daily throughout the season. The drivers that motivate having an individual staff can be multiple and very diverse and discussing their merits or lack thereof may be pointless. What is important is that this new reality is upon us and must be acknowledged and dealt with appropriately.

### Why Build Bridges?

Clubs and players may benefit more from building bridges (e.g., defining communication channels and establishing a coordination process) than building walls that may hinder internal and external efficiency. If we do not establish a proper communication channel with the players’ staff, this unintentionally works both ways [36]. Moreover, diluted personal responsibility regarding performance and health may ensue, resulting in decreased productivity from all involved (player, club, and personal staff practitioner) due to a lack of Distributed Situational Awareness [26, 27, 37]. For example, distinct staff members can influence how players experience each practice [72] due to personal experience, goals, roles, tasks, training, skills, schemata, and so on [26]. Situations, where professional overlap exists, may be prone to an intervention (e.g., testing procedure, injury prevention program, training program prescription, and training load management) being challenged by another practitioner [37]. This scenario may result (understandably) in a more concerned and anxious athlete [37].

Communication may “make or break” an organization’s effectiveness [36]. Fostering Distributed Situational Awareness allows for improved coordination and communication among distributed teams, which can help to prevent errors, reduce response times, and enhance overall performance [26, 27, 46, 73, 74]. By having a shared understanding of the situation, individuals and teams can work together more effectively and make more informed decisions [26, 27, 46, 73, 74]. What happens when something goes wrong (e.g., an injury or reinjury occurs)? Who would be accountable for that? Can injury rates of players be affected when having multiple advisors (there is a link between coach turnover and increased rates of muscle strain injuries [70])? Practitioners working individually for players may often put their job security above other considerations and may tend to act with this objective as a priority when things do not go as planned and consequently disclaim responsibility. Clubs and the players (through appropriate education) must be adequately sensible and conscient about the benefits and risks of this adoption.

In this line of thought, Brito and colleagues [75] raised attention to the risks associated with excessive exercise training-related (e.g., overreaching and overuse) and clinical-related (e.g., overdiagnosis and overmedicalization) interventions in current elite sports. These risks may increase parallel to the number of parties involved in athlete support if proper communication channels are not established (Fig. 1). So, the development of a joint working framework is warranted.

In this regard, if left unchecked, it may impact, not exclusively: (i) training load management and (ii) training programs optimization, and thus result, not solely, in: (i) excessive testing, (ii) early return to sports, and (iii) disruptions in nutrition and sleep education, habits, and shared meaning. Until an agreement on the context of players being concurrent under club and players’ staff supervision, the guidelines for improving the transition from elite clubs to national teams can support this initial process [20, 21, 25], namely with support in the following: (i) readiness to play, training status, and fitness (e.g., fatigue status, wellness, and physiological and performance testing), (ii) overall load management (e.g., training and competition load and internal and/or external load), (iii) specific (injury prevention and/or strength) programs (e.g., exercises, frequency, programming variables, and typical schedule), (iv) nutritional (e.g., supplementation) and sleep hygiene strategies, and (v) other aspects the player considers relevant (e.g., injury epidemiology, screening, and treatment information).

We acknowledge that different types of bridges may be needed and have different drawbacks. Notably, the differences between the groups one is trying to bridge may imply distinct forms of communication, interaction (e.g., from having lunch, invitation to watch sessions, or being “embedded” in specific training sessions), and information to share, customized to each club’s reality and each player’s personal staff qualities. How can someone build a bridge if he does not have access to the other side? Ideally, this should be a two-way process. With great power comes great responsibility (the importance of players’ education in understanding that personal staff should be a driver for putting everything together, as well).

### Best Practices Around Player Performance Management: Load, Training, and Monitoring

The importance of quantifying training load is extensively considered in the detailed frameworks of the majority of the performance, stress, strain, overuse-related, and injury prevention conceptual models in the scientific literature [76–78], and this significance has been extensively highlighted by several coaches and practitioners in specialized media. Furthermore, the relevance of fundamental training principles (e.g., overload and progression) in determining the individual player’s response is paramount [79–81]. Nevertheless, this new reality may indicate a disconnection between what is recommended and promoted in sports medicine, sports science literature, and specialized media, and what the involved parties are implementing. For example, although the club’s delivery of off-season training programs is a standard routine, players usually require outside services during this seasonal period. It is unclear whether the interests of the two parties are aligned. Although the player’s proactive action has several positive aspects in individual preparation [17], it also may have negative consequences if the stimulus received during this period is not considered at the club level when planning the precompetitive period [17, 20].

Another scenario where the absence of communication may strongly impact a player's training load management is during the competitive microcycle. The player’s chronic and acute exposure drives the planification at the club’s level. The latter, in particular, is one of the most robust individual variables that influence and modify short-term training prescription (48 h after the game) at the club [35, 43, 82–84]. As such, it is common in the days after the match that non-starter players receive an additional training stimulus for compensating the low or even absence of the specific neuromuscular and metabolic match stimulus [83]. On the other hand, starter players will undertake recovery interventions during the initial day(s) [83]. The days following a match provide a time window where some players may receive extra stimulus outside the club. Although it can be considered a “minimum amount of stimulus,” the individual thresholds that determine a minimal adaptive dose effectively, and how they interact with other types of stimuli, are not known [85]. However, manipulating the training stimulus within the post-match period should stick to each player’s specific needs, as it may be linked to the incidences of non-contact injuries [86].

In this regard, although there is the documented protective and performance enhancement effect of eccentric exercise and exposure to high-speed running in soccer players, more is not always better [75, 87, 88]. For example, a second daily session occurring outside the club (e.g., morning neuromuscular training session) may have a consequent impact on the intended adaptative response of the club’s next training session (e.g., afternoon). These may result in both sessions targeting the same physiological and performance determinants or vice/versa (e.g., involving a short interval between the two sessions creating a concurrent stimulus) [89–91]. Both situations may disadvantage the player’s preparation if the big picture (overall training plan) is not considered: double daily sessions may result in different endocrine, perceptual, and neuromuscular states than when performing single sessions [92]. Recovery from previous training sessions and “readiness” for subsequent training sessions may affect the magnitude of the training response incurred [93].

Independently of the season moment, if we fail to consider the stimulus outside the club settings, this may result in incorrect training load data-driven decision-making (from the club and the individual staff backing up each player), and well-informed decisions will be at risk [20, 35]. For example, let us imagine that this week, the player performed a light morning session designed to allow full recovery from the match (Tuesday, 48 h post-match, with the next session planned for Wednesday afternoon and Monday being day-off). Considering this schedule, the player performs an afternoon hard gym-based training session outside the club. However, the player does not know the overall weekly plan (and the club from the player), with two consecutive hard technical–tactical training sessions programmed for the Wednesday and Thursday afternoons. Nevertheless, the player also performed an additional session on Wednesday morning without knowing the coaching staff’s intentions. Now, let us imagine the player is doing more or less the same (having additional outside sessions 48 and 72 h post-match) consecutively during the last 2 weeks, without any exposure outside the club being consequently quantified. How can well-informed decisions occur in this context? Interestingly, during the men’s 2022 World Cup, FIFA developed a player app based on the input of the global representative of professional players, which made tracking data available to every player in the 2022 World Cup in Qatar [94]. Although there were concerns and difficulties in the interchangeability between locomotor movement variables collected with different tracking systems, efforts have been made to enable practitioners to combine and share data captured with varying systems of tracking [95, 96].

### Best Practices Around Player Health Management: Testing, Return to Sports, Sleep, and Nutrition Advice

Several testing approaches are applied in the club that may be replicated in the national team environment [21] and by the player’s staff (e.g., physiologist and strength and conditioning coach). The importance of muscular strength (e.g., lower limb posterior chain strength) for performance and injury prevention is recognized worldwide [15, 16, 88, 91]. Several performance and muscle strength profiling tools are now available at a low cost (e.g., force plates, hand-held dynamometers, linear position transducers). This easy access to technology may favor the reproduction (duplication of practice) or performance of new evaluation tasks that may not come up with additional information already existing at the club level [97, 98]. It may increase training and clinical-related features (e.g., overdiagnosis) [75]. Moreover, we have to consider that there may be cases where testing data may be erroneously collected and interpreted, i.e., due to a lack of familiarity with testing protocols, intra-individual variations associated with intra- and inter-rater reliability, measurement error that may result in incorrect interpretation of a true score change.

The return-to-play decisions after injuries are one of the most sensitive aspects of managing players’ performance and health. Coaches and players possess sufficient theoretical knowledge about soccer medicine [22]. Surveys from Loose et al. [22] and Buchheit et al. [99] suggested that an overwhelming majority of soccer players on the elite level may have the pretense to decide on medical issues on their own. Moreover, players often require external-to-the-club specialists and, in particular, intend to return to sport after injuries against the club physician’s recommendation [22, 99]. In case of little communication with the player’s staff, players may return to sport earlier than suggested by the club physician [22].

It is possible to observe performance enhancement (e.g., ergogenic aids) and injury prevention technologies and paradigms being promoted and acclaimed in the media and digital channels, with or without proper scientific support. Although modern players are more aware of the different dimensions of performance support, they are also more vulnerable to “noise” (disseminated primarily via unqualified social media) around performance optimization, with greater susceptibility in young and adult players [100–102]. This deliberate or inadvertent dis/misinformation in a fast-moving, result-dependent environment can make less informed players and practitioners open to persuasion by the advertisement of those who gain a commercial benefit (e.g., social media influencers and manufacturers resulting in inappropriate or inadequate Situational Awareness transactions) [28, 102, 103]. For example, players may be susceptible to experience an untested therapy that, although attractive because it “does not cause any harm,” has unevaluated long-term implications and a lack of evidence [104]. Moreover, players must be aware that subtle changes in their habits may affect sleep quality and their readiness to train in the following days [105, 106]. Also, there may be (or soon will be) an additional actor in a soccer team, i.e., wearable tech. How much and how often is a player using wearable tech to get feedback on training, sleep, diet, etc., etc., and how powerful (e.g., reliable) and effective is this feedback (e.g. limited interpretation of automated feedbacks) to the player concerning input from other human sources?

Furthermore, we should not exclude the possibility of someone from the players’ staff acting outside his scope of practice (e.g., nutritional and/or sleep advice provided by a non-nutritionist or a non-sleep specialist) [100]. Significantly, supplementation, without a nutrient deficiency, may negatively impact a player’s performance and health [103, 107]. Although outside the scope of this manuscript, the assessment methods of Distributed Situational Awareness [28] and its measurement could be considered a required organizational practice.

## Conclusion

We understand that it is impossible to quantify and control everything that may influence players’ performance and health, and this process is not as simple as it may seem. However, efforts must be implemented to clarify and certify complete management of the players’ performance and health environment, where the existence and impact of this personal staff are considered. Although the positive aspects of possessing a personalized support team can be easily named, if a proper communication channel is not established, this may unintentionally work both ways; just letting the processes into the hand of chance will not be the best way to proceed. This emerging reality calls for enhanced communication and data sharing across all the elements that provide players performance support; in other words, connecting the teams inside the “team”, the player’s personalized support team, with the club’s team. The final goal would be to put personal agendas aside and try to get the balance right and better manage a player-centered process.
